# High-Resolution Ultrasonographic Morphometry of the Superficial and Deep Peroneal Nerves in Healthy Young Adults: Reference Values From an Indian Cohort

**DOI:** 10.7759/cureus.114328

**Published:** 2026-08-11

**Authors:** Sanjula Singh, Garima Sehgal, Nikhil Aggarwal, Archana Rani, Anita Rani, Jyoti Chopra

**Affiliations:** 1 Anatomy, Maharani Laxmibai Medical College, Jhansi, IND; 2 Anatomy, King George's Medical University, Lucknow, IND; 3 Anatomy, Army College of Medical Sciences, New Delhi, IND

**Keywords:** body mass index, cross-sectional area, deep peroneal nerve, morphometry, superficial peroneal nerve, ultrasonography

## Abstract

Introduction

Peripheral nerve ultrasonography is an important diagnostic modality for evaluating nerve pathologies. Cross-sectional area (CSA) and perimeter measurements are key parameters for diagnosis and clinical management. The deep peroneal nerve (DPN) and superficial peroneal nerve (SPN) are prone to entrapment and traumatic injuries, yet normative morphometric data for these nerves in healthy individuals are limited. Understanding their relationship with anthropometric parameters such as body mass index (BMI) may help refine diagnostic criteria and guide procedures, including nerve blocks and surgical interventions.

Aim

The study aimed to assess the morphometric characteristics of the DPNs and SPNs using high-resolution ultrasonography (HRUS) in healthy young adults and to evaluate their association with BMI.

Materials and methods

This cross-sectional observational study was conducted in the Department of Anatomy, King George's Medical University, Lucknow, India, from September 2018 to August 2019. One hundred first-year medical students (50 males, 50 females; age 17-25 years) were included after informed consent. HRUS was performed using a linear array transducer (6-13 MHz) to measure CSA, perimeter, and depth from skin (DFS) of bilateral DPN and SPN at predetermined anatomical sites. The DPN was examined at the fibular neck and 3 cm proximal to the superior extensor retinaculum. The SPN was evaluated at the fibular neck, at the distal one-third of the lateral leg before piercing the deep fascia, and 5 cm above the lateral malleolus. Statistical analysis was performed using SPSS version 21.0 (IBM Corp., Armonk, NY). Paired t-tests assessed bilateral differences, independent t-tests evaluated gender differences, and one-way analysis of variance (ANOVA) compared BMI categories. Significance was set at p<0.05.

Results

The mean CSA of DPN was 7.90±1.70 mm² at site 1 (fibular neck) and 6.30±1.60 mm² at site 2 (3 cm proximal to the superior extensor retinaculum), with perimeter values of 8.88±1.44 mm and 7.61±1.56 mm, respectively. For SPN, mean CSA values were 8.40±1.80 mm² at site 1 (fibular neck), 6.30±1.60 mm² at site 2 (distal one-third of the lateral leg before piercing the deep fascia), and 5.90±1.40 mm² at site 3 (5 cm above the lateral malleolus), with perimeter measurements of 9.29±1.61 mm, 7.60±1.64 mm, and 7.28±1.53 mm, respectively. Significant bilateral differences were observed, with right-sided nerves larger than left-sided nerves (p<0.001). DPN CSA and perimeter were significantly greater in males than females (p<0.05), whereas SPN showed variable gender differences across sites. DFS measurements indicated both nerves were significantly deeper in females (p<0.001). No statistically significant association was found between nerve dimensions and BMI categories (p>0.05).

Conclusion

This study provides reference values for DPN and SPN morphometry in healthy young Indian adults. The observed bilateral asymmetry and gender differences highlight the need to consider these factors during ultrasound evaluation. The absence of significant BMI correlation suggests that nerve dimensions in healthy individuals are largely independent of BMI. These findings contribute normative data useful for diagnosing peroneal neuropathies and improving ultrasound-guided interventions.

## Introduction

The superficial and deep peroneal (fibular) nerves are the two terminal branches of the common peroneal nerve, which itself arises from the sciatic nerve in the popliteal fossa. The common peroneal nerve winds around the fibular head and then divides into the superficial and deep branches [1,2]. The superficial peroneal nerve (SPN) descends in the lateral compartment of the leg, coursing between the fibularis (peroneus) longus and brevis muscles and the extensor digitorum longus, before piercing the crural fascia in the distal third of the leg to enter the subcutaneous tissue [2]. It innervates the lateral compartment muscles (fibularis longus and brevis) and provides cutaneous sensory innervation to the anterolateral leg and most of the dorsum of the foot [3]. The deep peroneal nerve (DPN) travels in the anterior compartment alongside the anterior tibial artery. It supplies motor branches to the tibialis anterior, extensor digitorum longus, extensor hallucis longus, and extensor digitorum brevis muscles, and it provides sensory innervation to the first web space between the great toe and second toe [4,5]. At the ankle, the DPN passes under the extensor retinaculum through the anterior tarsal tunnel before dividing into medial and lateral branches in the foot. The precise anatomical course of these nerves can vary between individuals, but their superficial locations make them amenable to high-resolution imaging [2,6].

Clinically, injuries or entrapments of the peroneal nerves produce distinct syndromes. Compression of the common peroneal nerve at the fibular head causes weakness in ankle dorsiflexion and foot eversion (often manifesting as “foot drop”) with sensory loss over the dorsum of the foot [7]. Isolated superficial peroneal neuropathy -- sometimes observed in dancers, athletes, or after trauma -- typically occurs at the fascial exit point in the distal leg. Patients with SPN entrapment typically present with pain or paresthesia along the anterolateral leg and dorsum of the foot, while foot eversion is usually preserved because the motor branches arise proximal to the site of fascial entrapment. Deep peroneal neuropathy under the extensor retinaculum (“anterior tarsal tunnel syndrome”) typically presents with sensory loss in the first web space and weakness of toe extensor muscles, which may be subtle but can lead to reduced toe clearance or drop of the great toe during gait. In sum, SPN injury spares great toe extension and dorsiflexion but impairs foot eversion, whereas DPN injury spares eversion but can affect toe and ankle dorsiflexion [1]. These clinical distinctions underscore the need for detailed anatomical and functional understanding of each branch.

High-resolution ultrasonography (HRUS) has emerged as a key diagnostic tool for peripheral nerve disorders, especially those affecting superficial nerves. Ultrasound reliably visualizes peripheral nerves and can detect both acute and chronic changes such as swelling, fascicular enlargement, and perineurial thickening. The SPN and DPN, being subcutaneous or just below the fascia in their distal courses, are well-depicted on ultrasound. Indeed, ultrasound can trace the entire course of the SPN and DPN in the leg and foot, identify sites of compression or entrapment, and visualize lesions (e.g., neuromas and ganglia) that may impinge on these nerves. Sonographic studies report that SPN abnormalities (such as scar encasement, neuroma, or laceration) are readily observed, with ultrasound showing high sensitivity for detecting nerve pathology [1,4]. Importantly, ultrasound can aid in clinical diagnosis by correlating nerve morphology with patient symptoms, supplementing nerve conduction and electromyography studies. Thus, HRUS serves as an ideal modality for evaluating peroneal neuropathies.

Since nerve pathology often produces focal enlargement of the nerve, quantification of cross-sectional area (CSA) has become a standard ultrasound metric. Numerous recent studies have established normative CSA values for peripheral nerves, acknowledging that these values can vary by demographic factors [5-7]. For example, Hsieh et al. found that peroneal nerve CSAs in a healthy Taiwanese cohort were generally smaller than those reported in Western populations, and they observed that CSA correlated positively with weight and body mass index (BMI) [6]. Similarly, Bae and An reported normative CSA values for multiple lower-extremity nerves in healthy Korean adults, finding significant correlations between peroneal nerve CSA and height, weight, and BMI (with larger nerves in males) [5]. In an Indian population, Sindhu et al. provided CSA reference values for major limb nerves and noted that nerve size was significantly greater in men and positively correlated with body weight [7]. These findings suggest that ethnicity, gender, and anthropometric factors influence nerve size, and they underscore the need for population-specific reference ranges when interpreting ultrasound.

Despite these advances, major gaps remain in the literature on the SPNs and DPNs. Most ultrasound studies have focused on the common peroneal nerve at the fibular head or on peripheral nerves of the upper limb, leaving fewer dedicated data for the SPN and DPN branches. Normative ultrasound data for the SPNs and DPNs (at multiple levels bilaterally) are limited, particularly for South Asian and Indian populations. Moreover, existing studies have shown mixed results on how factors such as age, BMI, and sex affect lower limb nerve CSAs [5,6]. In some reports correlations with BMI are strong, while others find minimal or no effect, highlighting uncertainty about these associations. While the anatomy and entrapment syndromes of the peroneal nerves are well described, more research is needed to establish normative sonographic parameters for SPN and DPN across diverse populations and to clarify how demographic factors influence nerve morphology.

Hence, given the paucity of detailed bilateral reference data for both DPN and SPN in young, healthy adults and the conflicting evidence regarding BMI correlations, this study aimed to fill critical gaps by performing a cross-sectional ultrasonographic evaluation of DPN and SPN morphometry at standardized sites in 100 asymptomatic medical students. We measured CSA, perimeter, and depth from skin (DFS) bilaterally, analyzed inter-site and side-to-side differences, and assessed the impact of BMI on nerve dimensions. The resulting normative database seeks to enhance diagnostic precision for peroneal neuropathies and guide ultrasound-assisted interventions around the ankle.

## Materials and methods

Study design and participants

A cross-sectional observational study was conducted in the Department of Anatomy at King George's Medical University, Lucknow, India, from September 2018 to August 2019. Ethical approval for the study was obtained from the Institutional Ethics Committee, King George's Medical University, Lucknow, India (Approval No. 857/Ethics/R.Cell-18; Reference Code: 93rd ECM IIB Thesis/P6, dated 14 November 2018). Written informed consent was obtained from all participants before enrolment. One hundred healthy first-year MBBS and BDS students (50 males, 50 females; age 17-25 years) were recruited after informed consent. Exclusion criteria included any history or clinical signs of peripheral neuropathy, musculoskeletal disorders, systemic illnesses (e.g., diabetes), prior lower limb surgery, or trauma.

Anthropometric measurements

Height (cm) and weight (kg) were measured using a stadiometer and digital scale, respectively, and BMI (kg/m²) was calculated for each participant. Participants were categorized as underweight (<18.5), normal (18.5-24.9), or overweight/obese (≥25) based on BMI values.

Ultrasound equipment and settings

HRUS was performed with an Esaote MyLab™ 40 system (Esaote Europe, Genoa, Italy) equipped with a 6-13 MHz linear transducer. The machine was calibrated daily, and gain, depth, and focus were adjusted to optimize nerve visualization.

Nerve imaging protocol

Participants were positioned supine with legs extended. Ultrasound gel was applied to predetermined anatomical landmarks. Nerves were identified by their characteristic "honeycomb" echotexture in transverse section, described as dark punctuate areas (fascicle groups) surrounded by hyperechoic bands (perineurium). In longitudinal section, nerves appeared as parallel hypoechoic and hyperechoic lines. 

SPN

**Figure 1:**
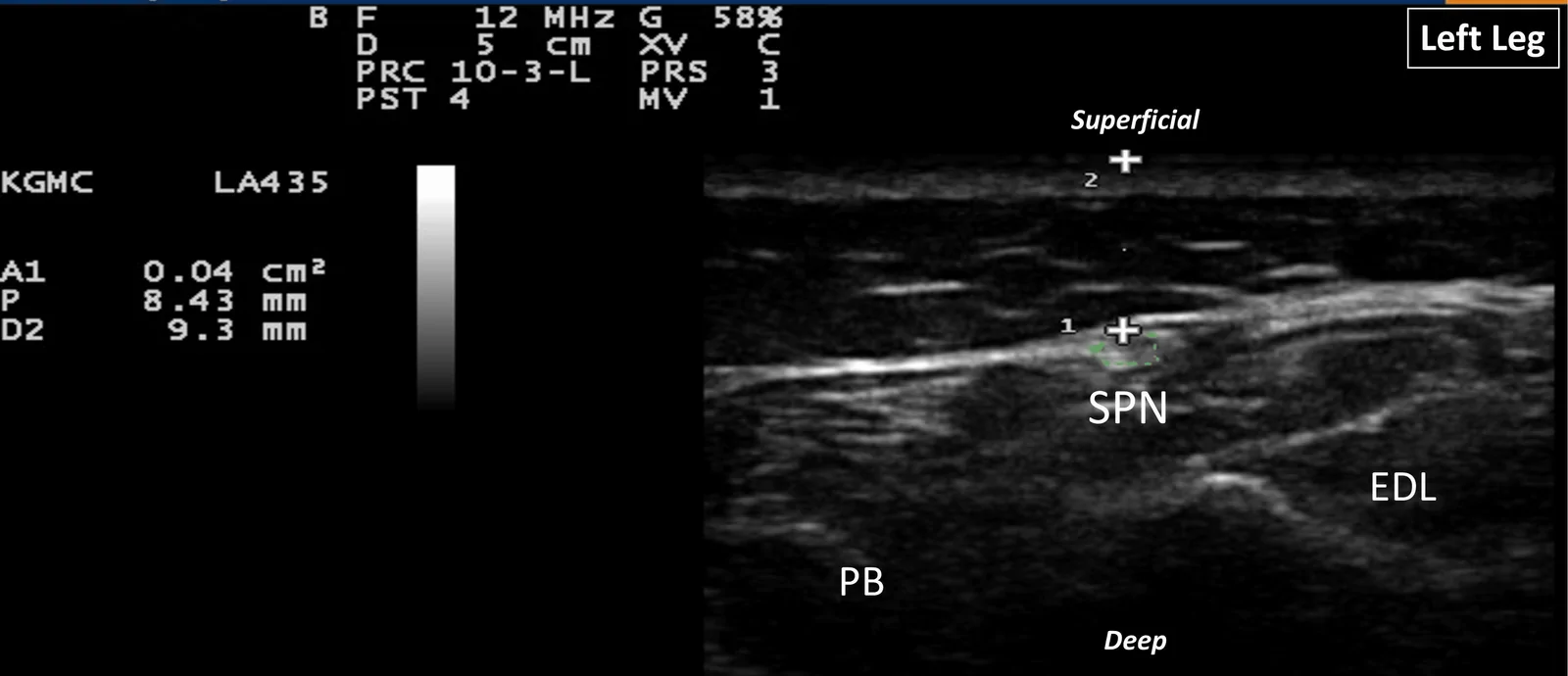
Ultrasonographic image of left leg showing the superficial peroneal nerve before piercing the deep fascia. EDL, extensor digitorum longus; PB, peroneus brevis; SPN, superficial peroneal nerve.

**Figure 2:**
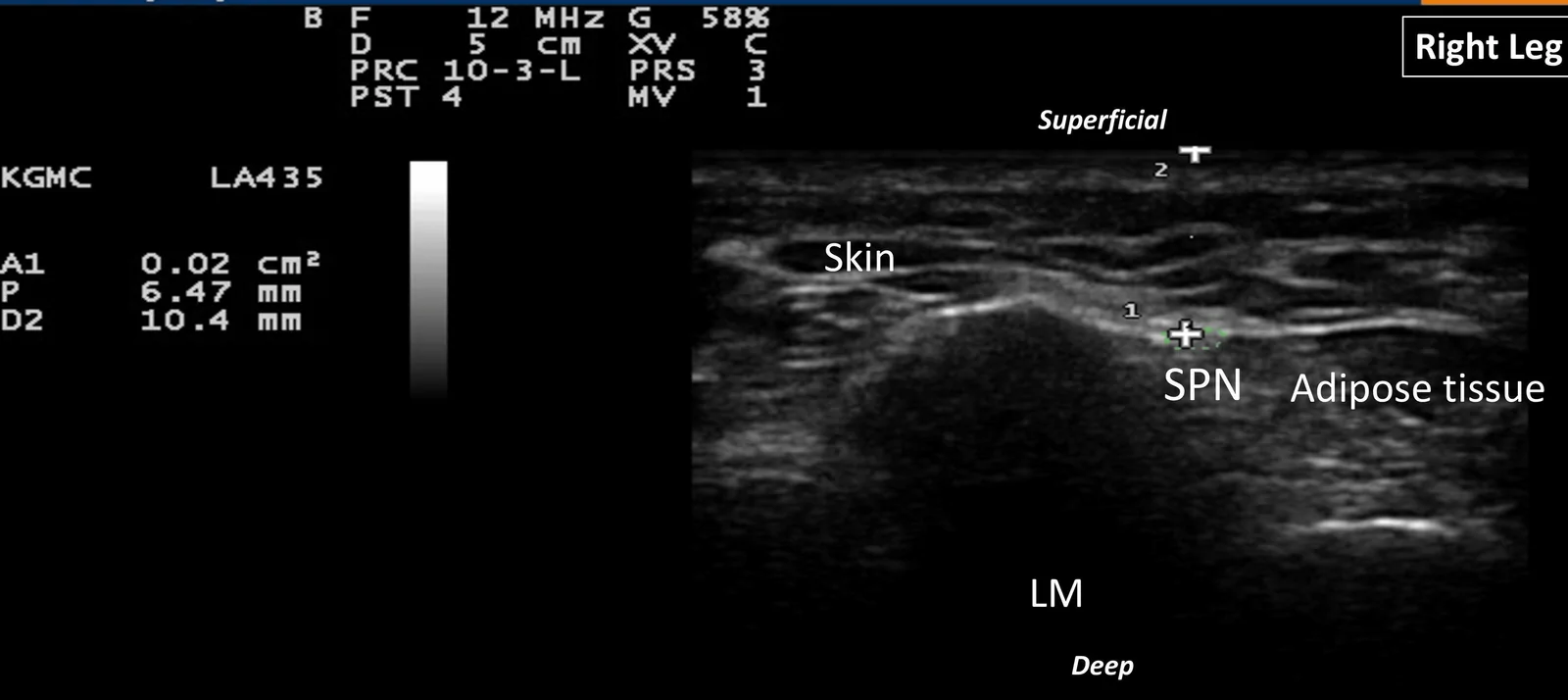
Ultrasonographic image of right leg showing the superficial peroneal nerve at 5 cm above the lateral malleolus. A, artery; f, fibula; LM, lateral malleolus; SPN, superficial peroneal nerve; t, tibia.

**Figure 3:**
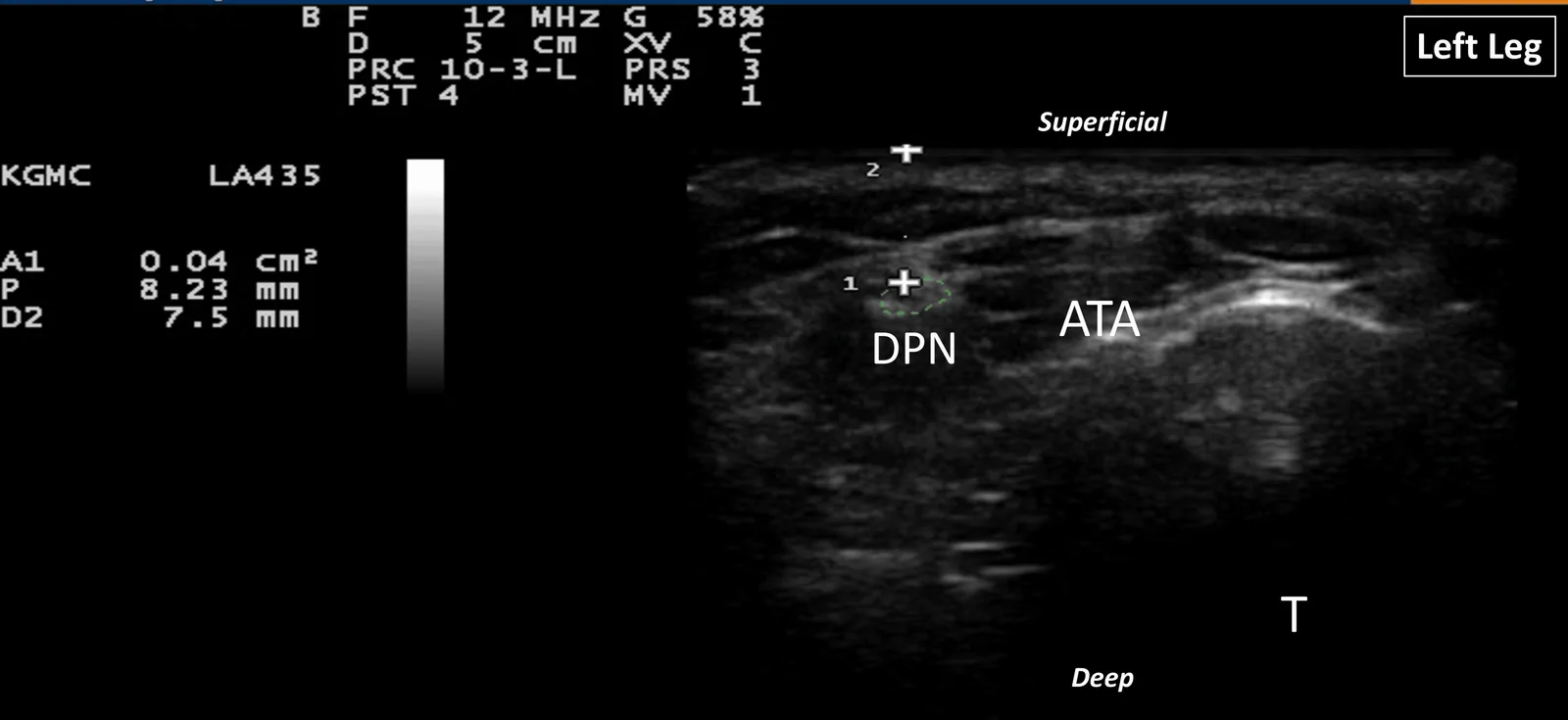
Ultrasonographic image of left leg showing the deep peroneal nerve at 3 cm above the superior extensor retinaculum. TA, anterior tibial artery; DPN, deep peroneal nerve; T, tibia.

Three measurement sites were examined: (1) Site 1: At its origin from the common peroneal nerve at the fibular neck, (2) Site 2: At the distal third of the lateral leg before piercing the deep crural fascia (approximately 10-12.5 cm above the lateral malleolus) (Figure 1), and (3) Site 3: 5 cm above the lateral malleolus in the subcutaneous plane, piercing the fascia (Figure 2).

DPN

Two measurement sites were assessed: (1) Site 1: At its origin from the common peroneal nerve at the fibular neck and (2) Site 2: 3 cm proximal to the superior extensor retinaculum, adjacent to the anterior tibial artery (Figure 3).

For each nerve, the following parameters were measured bilaterally on transverse images using the trace method, carefully avoiding the hyperechoic perineurial rim: (1) CSA: Direct tracing around the nerve fascicles, (2) Perimeter: Length of the traced nerve outline, and (3) DFS: Distance from the skin surface to the center of the nerve.

Three measurements were obtained for each parameter at each site; the mean value was recorded. All measurements were performed by a single experienced observer to ensure consistency.

Statistical analysis

Data were analyzed using SPSS version 21.0 (IBM Corp., Armonk, NY). Continuous variables are presented as mean ± SD. Paired t-tests compared right versus left measurements; independent t-tests compared male versus female values. One-way analysis of variance (ANOVA) assessed differences across BMI categories for each nerve. Statistical significance was defined as p<0.05. Highly significant results were considered when p<0.001. 

## Results

One hundred healthy volunteers (50 males, 50 females) aged 17-25 years were included in this study. The mean age was 19.65±1.44 years, with a mean height of 163.65±8.19 cm and a mean weight of 61.72±10.60 kg. The calculated mean BMI was 22.94±3.27 kg/m², indicating a predominantly normal-weight population (Table 1).

**Table 1:** Demographic characteristics of study population (n=100) Data are presented as mean ± standard deviation (SD) for continuous variables and number (N) and percentage (%) for categorical variables. BMI, body mass index.

Parameter	Mean±SD	Range
Age (years)	19.65±1.44	17-25
Height (cm)	163.65±8.19	150-184
Weight (kg)	61.72±10.60	40-91
BMI (kg/m²)	22.94±3.27	15.62-31.11
Male:female ratio	1:1	-

Based on BMI classification, 6 (6%) subjects were underweight, 72 (72%) had normal weight, 19 (19%) were overweight, and 3 (3%) were obese. This distribution demonstrates minimal representation of extreme BMI categories, which supports the suitability of the dataset for generating stable reference values.

The SPN was successfully identified and measured at all three predetermined anatomical sites in all participants, as shown in Table 2. It demonstrated the expected morphometric pattern of proximal-to-distal tapering, with the largest dimensions at its origin (Site 1) and smallest at the distal site (Site 3). The CSA of the nerve decreased from 8.40±1.80 mm² proximally to 5.90±1.40 mm² distally. Significant intersite differences were observed for all measured parameters across sites (p<0.001), which supports the need for site-specific reference values.

**Table 2:** Morphometric parameters of superficial peroneal nerve at different sites Statistical tests used: One-way analysis of variance (ANOVA) Site 1: At fibular neck; Site 2: Distal 1/3 of lateral leg; Site 3: 5 cm above lateral malleolus Values are expressed as mean ± SD unless otherwise specified. Statistical significance was defined as p<0.05. Highly significant results were considered when p<0.001. CSA, cross-sectional area; DFS, depth from skin; SD, standard deviation.

Site	Parameter	Min	Max	Mean±SD	F-value	p-value
Site 1	CSA (mm²)	5.0	12	8.40±1.80	128.488	<0.001
	Perimeter (mm)	5.21	12.53	9.29±1.61	91.416	<0.001
	DFS (mm)	6.4	11.2	8.94±1.09	11.560	<0.001
Site 2	CSA (mm²)	4.0	10	6.30±1.60		
	Perimeter (mm)	4.25	11.7	7.60±1.64		
	DFS (mm)	5.2	10.3	8.11±1.09		
Site 3	CSA (mm²)	3.0	10	5.90±1.40		
	Perimeter (mm)	4.10	10.52	7.28±1.53		
	DFS (mm)	5.0	9.4	7.73±4.18		

The DPN, being the smaller terminal branch of the common peroneal nerve, showed correspondingly smaller morphometric values compared with other peripheral nerves. Table 3 describes all the parameters, as it was consistently visualized at both measurement sites. The nerve demonstrated significant reduction from Site 1 (7.90±1.70 mm²) to Site 2 (6.3±1.60 mm²), along its course through the anterior compartment muscles. The nerve showed statistically significant intersite variations similar to the SPN. 

**Table 3:** Morphometric parameters of deep peroneal nerve at different sites Statistical tests used: One-way analysis of variance (ANOVA) Site 1: At fibular neck; Site 2: 3 cm above superior extensor retinaculum Values are expressed as mean ± SD unless otherwise specified. Statistical significance was defined as p<0.05. Highly significant results were considered when p<0.001. CSA, cross-sectional area; DFS, depth from skin; SD, standard deviation.

Site	Parameter	Min	Max	Mean±SD	F-value	p-value
Site 1	CSA (mm²)	4.0	12	7.90±1.70	9.239	<0.001
	Perimeter (mm)	5.15	11.9	8.88±1.44	8.484	<0.001
	DFS (mm)	6.50	11.9	9.21±1.18	8.593	<0.001
Site 2	CSA (mm²)	3.0	10	6.30±1.60		
	Perimeter (mm)	4.24	11.35	7.61±1.56		
	DFS (mm)	6.40	10.9	8.35±0.79		

The bilateral comparison revealed significant right-left asymmetries in nerve parameters (Table 4). Most notably, the SPNs and DPNs consistently showed larger CSAs on the right side.

**Table 4:** Right vs. left side comparison of peroneal nerves Statistical tests used: Paired t-test; significance level: p<0.05 Values are expressed as mean ± SD unless otherwise specified. Statistical significance was defined as p<0.05. Highly significant results were considered when p<0.001. CSA, cross-sectional area; SD, standard deviation.

Nerve	Parameter	Right side	Left side	t-value	p-value
Superficial peroneal	Overall CSA (mm²)	7.2±2.1	6.6±1.8	4.515	<0.001
	Overall perimeter (mm)	8.50±1.72	7.62±1.80	-6.841	<0.001
Deep peroneal	Overall CSA (mm²)	7.6±1.8	6.6±1.6	7.346	<0.001
	Overall perimeter (mm)	8.81±1.37	7.69±1.68	-8.075	<0.001

Gender-based analysis revealed significant sexual dimorphism in peripheral nerve morphometry, with males generally showing larger CSAs while females demonstrated greater subcutaneous depth (Table 5). Males consistently demonstrated larger CSAs across most nerve sites, for both SPNs and DPNs.

**Table 5:** Gender comparison of peroneal nerve parameters Statistical tests used: One-way analysis of variance (ANOVA) Values are expressed as mean ± SD unless otherwise specified. Statistical significance was defined as p<0.05. Highly significant results were considered when p<0.001. CSA, cross-sectional area; DFS, depth from skin; SD, standard deviation.

Peripheral nerve	CSA (mm²)					Perimeter (mm)					DFS (mm)				
	Female		Male		p-value	Female		Male		p-value	Female		Male		p-value
	Mean	SD	Mean	SD		Mean	SD	Mean	SD		Mean	SD	Mean	SD	
Superficial peroneal nerve															
Site 1	8.3	1.8	8.4	1.9	0.513	9.40	1.54	9.18	1.67	0.327	9.04	1.13	8.85	1.05	0.217
Site 2	6.0	1.6	6.6	1.6	0.011	7.73	1.62	7.48	1.66	0.290	8.39	0.97	7.84	1.13	<0.001
Site 3	5.7	1.5	6.2	1.3	0.014	7.35	1.48	7.21	1.58	0.523	7.93	0.89	7.84	5.85	0.723
Site-wise (ANOVA)	F=75.301; p<0.001		F=55.823; p<0.001			F=49.638; p<0.001		F=42.199; p<0.001			F=49.583; p<0.001		F=2.769; p=0.064		
Deep peroneal nerve															
Site 1	7.5	1.7	8.2	1.6	0.001	8.91	1.44	8.85	1.45	0.775	9.53	1.20	8.90	1.08	<0.001
Site 2	6.1	1.7	6.6	1.5	0.015	7.88	1.54	7.34	1.53	0.014	8.48	0.79	8.21	0.77	0.015
Site-wise (ANOVA)	F=6.021; p<0.001		F=7.331; p<0.001			F=4.900; p<0.001		F=7.165; p<0.001			F=7.241; p<0.001		F=5.175; p<0.001		

The BMI-based analysis revealed minimal associations between BMI and nerve morphometric parameters, for either SPNs or DPNs (Table 6). 

**Table 6:** BMI-based comparison of nerve parameters Statistical tests used: One-way analysis of variance (ANOVA) Values are expressed as mean ± SD unless otherwise specified. Statistical significance was defined as p<0.05. Highly significant results were considered when p<0.001. BMI, body mass index; CSA, cross-sectional area; DFS, depth from skin; SD, standard deviation.

Peripheral nerve	CSA (mm²)								Perimeter (mm)							DFS (mm)						
	Underweight		Normal		Overweight + obese			p-value	Underweight		Normal		Overweight + obese		p-value	Underweight		Normal		Overweight + obese		p-value
	Mean	SD	Mean	SD	Mean	SD			Mean	SD	Mean	SD	Mean	SD		Mean	SD	Mean	SD	Mean	SD	
Superficial peroneal nerve																						
Site 1	7.3	1.8	8.3	1.8	8.8	1.8	0.047		8.61	2.07	9.33	1.59	9.35	1.54	0.320	9.08	1.11	8.94	1.12	8.92	1.01	0.903
Site 2	5.5	1.7	6.4	1.6	6.4	1.7	0.195		7.49	1.71	7.60	1.62	7.64	1.71	0.962	8.26	1.03	8.16	1.08	7.94	1.14	0.457
Site 3	5.5	1.5	5.9	1.4	6.0	1.3	0.553		7.36	1.35	7.24	1.60	7.41	1.32	0.801	7.76	1.04	7.85	4.88	7.34	1.01	0.777
Site-wise (ANOVA)	F=4.788; p<0.001		F=88.201; p<0.001		F=38.309; p<0.001				F=1.872; p<0.001		F=69.476; p<0.001		F=21.081; p<0.001			F=4.713; p<0.001		F=5.177; p<0.001		F=24.943; p<0.001		
Deep peroneal nerve																						
Site 1	7.2	1.6	8.0	1.7	7.7	1.7	0.215		8.76	1.01	8.97	1.47	8.62	1.45	0.350	9.41	1.27	9.19	1.17	9.22	1.22	0.828
Site 2	5.8	1.6	6.4	1.6	6.4	1.6	0.528		7.60	1.58	7.65	1.52	7.50	1.68	0.852	8.37	0.73	8.30	0.82	8.48	0.72	0.454
Site-wise (ANOVA)	F=71.989; p<0.001		F=8.366; p<0.001		F=3.557; p=0.001				F=2.146; p<0.001		F=7.522; p<0.001		F=3.370; p<0.001			F=2.466; p<0.001		F=7.435; p<0.001		F=3.514; p<0.001		

All intersite differences in nerve dimensions for both nerves were highly significant (p<0.001). The SPN showed the most pronounced proximal-to-distal size reduction, while the DPN demonstrated more gradual changes. Bilateral asymmetry was consistently observed, with right-side predominance in both nerve groups. Gender-specific differences were significant for depth measurements, with female nerves positioned more superficially despite having smaller CSAs.

## Discussion

The present study provides one of the most detailed ultrasonographic evaluations of the SPN and DPN in healthy young adults, offering bilateral, multi-level morphometric reference values for a South Asian population. The results revealed clear and consistent trends in nerve dimensions along their anatomical course and patterns influenced by sex, laterality, and anthropometric variables.

A key finding was the progressive decrease in nerve size from proximal to distal segments for both branches of the common peroneal nerve. The SPN demonstrated a reduction in CSA from 8.40±1.80 mm² at the fibular neck to 5.90±1.40 mm² at 5 cm above the lateral malleolus. Similarly, the DPN decreased from 7.90±1.70 mm² proximally to 6.30±1.60 mm² distally. This proximal-to-distal tapering pattern aligns with expected anatomical redistribution of fascicles as nerves descend and give off muscular and cutaneous branches. These results reinforce the importance of site-specific normative values when interpreting sonographic nerve measurements.

The study also demonstrated significant right-left differences, with the right side consistently showing slightly larger CSA and perimeter values for both nerves. Although the magnitude of asymmetry was modest, this trend may reflect physiological adaptations associated with limb dominance, as described for other peripheral nerves [7-12]. The presence of such asymmetry highlights the need for clinicians to interpret ultrasound findings using side-to-side comparison within the same individual, especially when evaluating suspected entrapment or neuropathy.

Sex-based differences were also prominent in our dataset. Males exhibited significantly larger CSAs at most sites for both SPN and DPN, consistent with prior reports demonstrating larger peripheral nerves in men, likely due to greater limb girth, muscle mass, and hormonal influences on neural development [5,13]. In contrast, females showed greater DFS across nearly all sites, reflecting differences in subcutaneous fat distribution. These findings have practical implications for both diagnostic and interventional procedures, as deeper nerve positioning may influence needle trajectory during ultrasound-guided nerve blocks, particularly at the ankle.

BMI-related findings revealed no statistically significant association between BMI categories and morphometric parameters for either nerve. This contrasts with some earlier studies that reported positive correlations between nerve CSA and body habitus [5,6,9]. The minimal BMI effect observed in the present study may be explained by the narrow BMI range and the young age of the participants, suggesting that within healthy young adults, nerve size remains largely stable across BMI categories. This finding strengthens the utility of the present reference values for clinical interpretation in similar populations.

A major interpretative point of this study is the limited availability of published normative data for SPN and DPN, particularly at multiple anatomical levels and in bilateral measurements. Much of the existing literature focuses on the common peroneal nerve at the fibular head [8,9,11,13], with relatively few studies addressing its terminal branches in detail. As a result, direct comparison of our SPN and DPN measurements with previous work is constrained. Nonetheless, our proximal CSA values fall within the broader range reported for the common peroneal nerve, reflecting methodological concordance in ultrasonographic tracing and nerve identification techniques. The lack of extensive prior research on SPN and DPN not only limits comparative analysis but also underscores the novelty and clinical importance of the present dataset. A comparative summary of relevant studies is provided in Table 7 below. 

**Table 7:** Comparative summary of published studies on morphometry of the superficial and deep peroneal nerves, highlighting study design, population, key findings, and methodological differences, alongside the present study for direct comparison. Data presented as reported in the original studies. BMI, body mass index; CI, confidence interval; CPN, common peroneal nerve; CSA, cross-sectional area; DFS, depth from skin; DPN, deep peroneal nerve; L/R, left/right; SPN, superficial peroneal nerve; US, ultrasonography.

Author (year)	Study type and method	Population/location	N	Key findings	Method/definition notes
Peeters et al. (2004) [11]	US imaging, linear transducer	Belgium, single center	15	Visualized CPN at fibular head; BMI ↑ correlated with right CPN transverse diameter; no L/R differences in CSA	Measured diameters and “surface area” at fibular head; small N
Kerasnoudis et al. (2013) [13]	US imaging of multiple nerves	Germany, single center	75	Fibular (common peroneal) nerve CSA larger in males than females (popliteal level, p<0.034); CSA did not correlate with height	Used high-frequency US; CSA trace vs. variability metrics
Bedewi et al. (2018) [9]	US imaging of lower limb nerves	Saudi Arabia, single center	69	CP nerve at fibular head: mean CSA: 8.9±3.2 mm²; CSA correlated positively with age, weight, BMI; no R/L CSA difference reported	Measured 5 sites per leg; traced CSA; reported bilateral means
Bae and An (2022) [5]	US imaging of lower limb nerves	South Korea, single center	107	CSA measured at 12 lower-ext sites; CSA correlated with height/weight/BMI; larger in males at most nerves except SPN (distal calf) and CPN (fibular head)	Standardized protocol; unilateral measurements; full demographics
Fisse et al. (2021) [8]	Systematic review and meta-analysis	Various (multicenter data)	1001	Pooled CSA: common (fibular) nerve at fibular head 8.4 mm² (CI: 6.8–9.9); at popliteal 7.9 mm² (CI: 6.6–9.2); large heterogeneity in tibial nerve data	Aggregated 16 studies; focused on fibular, tibial, sural nerves
Kuga et al. (2021) [10]	US imaging (anatomical variants)	Japan, single center	100	Examined CSA at 10 sites; noted that fine branching causes CSA decreases proximally->distally up to ~2.5 mm² in fibular nerve; ankle circumference and age associated with some CSA values	Emphasized variation due to branching patterns
Present study (2025)	US imaging of SPN and DPN	India, single center	60	SPN CSA: ~8.4→5.9 mm² (proximal→distal); DPN: ~7.9→6.3 mm²; significant R>L side differences; males > females (larger CSA), females deeper nerves (DFS higher); BMI effect minimal	Bilateral US; direct trace measurement of CSA; standardized landmarks

The current study therefore contributes substantially to filling this gap by providing branch-specific, level-specific normative data. These measurements can aid clinicians in identifying pathological nerve enlargement, improving diagnostic accuracy in conditions such as SPN entrapment, anterior tarsal tunnel syndrome, traumatic neuropathies, and postsurgical complications. Additionally, the DFS measurements provide practical guidance for performing ultrasound-guided regional anesthesia, as nerve depth varies meaningfully across sex and anatomical level.

Overall, the present findings emphasize that SPN and DPN possess predictable morphometric patterns influenced by anatomical location, sex, and laterality but remain largely independent of BMI within healthy young adults. The scarcity of comparable reference data in the literature enhances the value of this study and positions it as a meaningful contribution to the normative database for lower-limb peripheral nerve ultrasonography.

Limitations

The study’s limitations include the relatively narrow age range (17-25 years) and homogeneous young adult sample, which restricts generalizability to older or pediatric populations where nerve dimensions may differ due to age-related or developmental factors. The sample also had a limited spectrum of BMI variation, with few obese participants, potentially constraining detection of BMI effects on nerve size. All measurements were performed by a single experienced observer, which ensured consistency but precluded assessment of inter-observer variability and introduced the possibility of systematic bias, although intra-rater reliability was confirmed. Another limitation lies in the restricted availability of published literature specifically evaluating the SPNs and DPNs. Most prior studies focus predominantly on the common peroneal nerve at the fibular head [8,9,11,13], leaving limited comparative data for SPN and DPN morphometry. This constrained the extent of cross-study comparison. However, this very gap also highlights the unique contribution of the present study, which provides comprehensive, branch-specific reference values at multiple anatomical levels. We measured CSA exclusively by direct tracing; alternative methods such as the ellipse formula or different imaging planes might yield slightly different results. Electrophysiological correlations (e.g., electromyography or nerve conduction studies) were not assessed, and the imaging protocol focused on the segment of the nerve under the crural fascia, without evaluation of more distal foot-level sites that could be clinically relevant in certain pathologies. Future research should include a broader age range, diverse BMI groups, multiple imaging examiners, and combined anatomical-functional assessments to validate and extend these normative values.

## Conclusions

This study provides normative sonographic data for the SPNs and DPNs, confirming that they taper distally and are generally symmetric but larger in males. The results mostly agree with the existing literature, though discrepancies (e.g., in side differences and BMI effects) highlight the importance of technique and population context. Future research should expand these norms to varied ages and ethnicities, investigate the impact of anatomical variants (as others have noted for lower limb nerves), and assess diseased states (e.g., diabetic neuropathy or entrapments) against this baseline. Ultimately, these insights may improve the accuracy of ultrasound in diagnosing peroneal neuropathies and guiding interventions such as nerve blocks or surgical decompression.
